# Coexisting Malignant Melanoma and Blue Nevus of the Uterine Cervix: An Unusual Combination

**DOI:** 10.1155/2012/986542

**Published:** 2012-09-16

**Authors:** David Parada, Karla B. Peña, Frances Riu

**Affiliations:** ^1^Departament de Anatomía Patològica, Hospital Universitari Sant Joan de Reus, Reus, 43201 Tarragona, Spain; ^2^Institut d'Investigació Sanitaria Pere Virgili (IISPV), Universitat Rovira i Virgili, Reus, Tarragona, Spain

## Abstract

Malignant melanoma (MM) and blue nevi of the uterine cervix are an extremely rare neoplasm, probably derived from embryologic migration of melanocytes from the neural crest. MM displays aggressive behavior with a poor prognosis. We report the case of a 76-year-old postmenopausal woman abnormal vaginal bleeding. She underwent a hysterectomy and bilateral salpingo-oophorectomy with paraaortic-iliac lymphadenectomy. Histopathological and immunohistochemical studies were consistent with the diagnosis of MM and blue nevi in the uterine cervix. Although it is extremely rare, this case suggests that MM of the uterine cervix should be considered in the differential diagnosis of undifferentiated neoplasm. Early diagnosis is essential in order to warrant a better prognosis, although there are no cases of cure described.

## 1. Introduction

 Primary malignant melanoma (MM) of the cervix represents an exceedingly uncommon neoplasia [1–6], representing less than 2% of cases of MM that affects the female genital tract [1, 2]. Based on the literature survey of uterine cervical MM, few cases are well documented [1]. Taylor and Tuttle reported the first well documented case in 1944 [7], but the concept of primary MM in uterine cervix was accepted after the description by Cid [8] of melanocytic cells in the cervix. Primary cervical uterine MM is considered as a very aggressive neoplasia [9].

 Our purpose is to report the case of a patient with uterine cervix malignant melanoma coexisting with blue nevi. To the best of our knowledge, this association has not been previously informed.

## 2. Case Report

A 76-year-old postmenopausal female patient visited the outpatient clinic with a six-month abnormal vaginal bleeding. She had a previous history of hypertension, dyslipidemia, osteoporoses, and rheumatic arthritis. On physical exploration, vaginal introit and uterine cervix were atrophies. An abdominal ultrasound was performed with uterine polyp compatible finding. An endometrial biopsy was carried out. After initial diagnostic, a chest X-ray, cystoscopy, and colonoscopy were performed and reported as normal. In addition, a complete revision of skin and eyes, to discard melanocytic lesions, was performed.

The patient was considered to be a candidate for exploratory laparoscopy and total hysterectomy, bilateral salpingo-oophorectomy, and iliac-paraaortic lymphadenectomy. Intrahospital evolution was unsatisfactory, and the patient stayed at the hospital for one month.

### 2.1. Pathology

#### 2.1.1. Endometrial Biopsy

**Figure 1:**
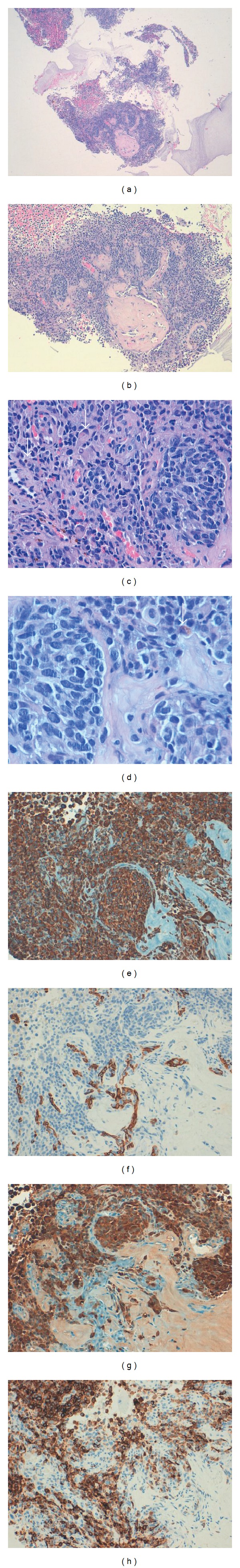
Endometrial biopsy. Histopathologic findings. (a, b, c, and d) Polypoid appearance. The tumor cells arranged in sheets or irregular nests, cytoplasm shows a granular eosinophilic pattern. A dark brown intracytoplasmatic pigment is present (white arrows). Immunohistochemical studies. (e, f, g, and h). S-100 expression, (f) cytokeratin positivity. (g, h). HMB45 and Melan A positivity in neoplastic cells, respectively.

Endometrial biopsy showed multiple fragments of neoplastic tissue with polypoid appearance (Figure 1). The tumor cells were arranged either in sheets or irregular confluent nests. Tumor cells were polygonal with granular eosinophilic cytoplasm. A fusiform-like malignant pattern was also observed. Occasionally, a dark brown intracytoplasmatic pigment was present (Figure 1). Immunohistochemical studies showed positivity of the neoplastic cells for vimentin, S-100 protein, HMB45, and Melan A. An interpretation of abnormal expression to cytokeratin AE1/AE3 was also seen (Figure 1). No normal endometrial tissue was present. The presumptive diagnosis was malignant Müllerian mixed tumor with melanocytic features; however, malignant melanoma could not be discarded.

#### 2.1.2. Hysterectomy and Bilateral Salpingo-Oophorectomy

**Figure 2:**
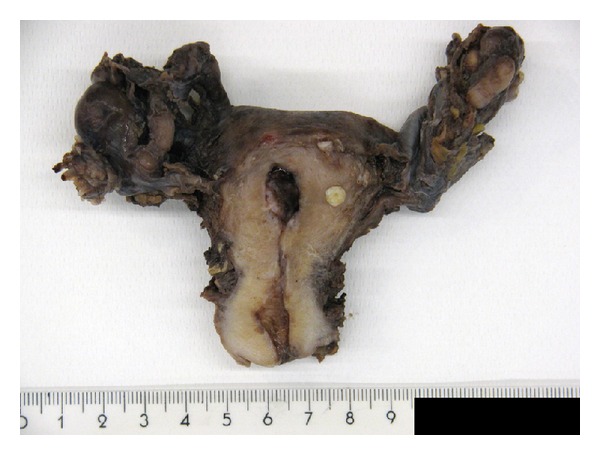
Surgical resection showing a polyp mass at uterine cavity (Note no other findings are evident).

Surgical specimen showed a polyp lesion at endometrial fund cavity (Figure 2). Two well-defined intramural nodular lesions (leiomyomas) were founded in myometrial wall. No other macroscopic findings were evident. The microscopic finding showed an endometrial polyp with no atypia. The entire endometrial cavity was embedding to histopathological analysis with no evidences of malignancies.

**Figure 3:**
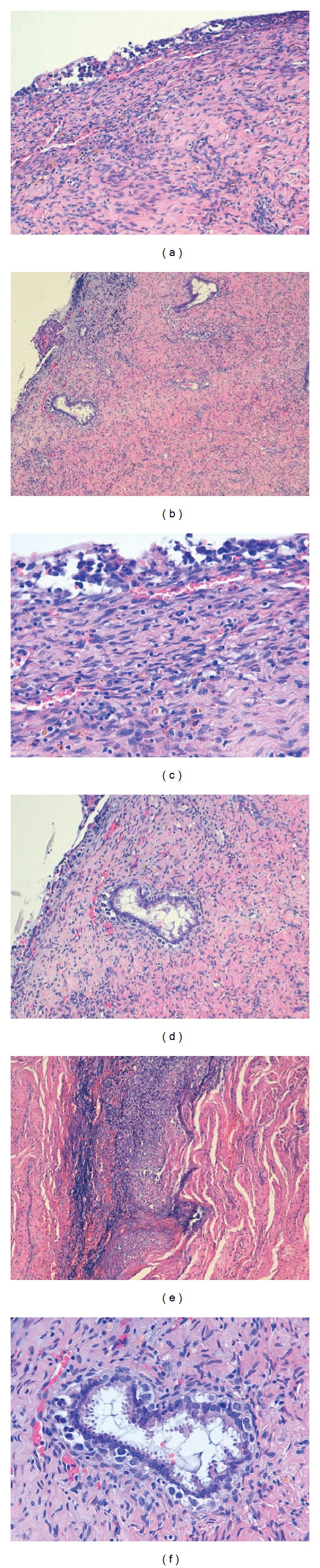
Uterine cervical malignant melanoma. (a, b, c, d, e, and f) Extensive epithelial denudation accompanied of atypical cells. The cells showed an epithelioid appearance. Pagetoid glandular extension and stromal invasion are present.

**Figure 4:**
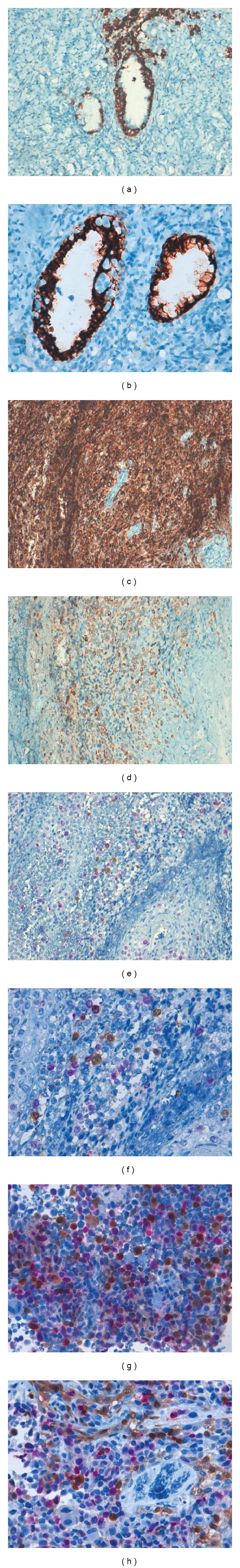
Uterine cervical malignant melanoma. Immunohistochemical studies. (a, b). S-100 and cytokeratin 7 expression. (c, d). HMB45 and Melan A positivity in neoplastic cells, respectively. (e, f, g, and h) CINtec Plus (Dual p16 + Ki-67), showing proliferate activity (red) and p16 (brown) positivity at nuclear and cytoplasm malignant cells.

Microscopically, uterine cervix showed extensive epithelial denudation accompanied of atypical cells forming ill-defined nest. The cells had an epithelioid appearance, broad cytoplasmic cells, poorly defined borders with pleomorphic nuclei and prominent nucleoli. Pagetoid glandular extension was observed. Stromal invasion was present (Figure 3). Immunohistochemical studies showed positivity of the neoplastic cells for vimentin, S-100 protein, HMB45, and Melan A. Cytokeratin AE1/AE3, CAM5.2 and cytokeratin 7 were negatives (Figure 4). CINtec Plus (Dual p16 + Ki-67) was performed in both endometrial biopsy and cervical lesion showing elevate proliferate activity. p16 positivity was observed at nuclear and cytoplasm malignant cells in both cases (Figure 4). Lymph node involvement was identified in one of the eleven isolated lymph nodes (isolated tumor cells, showing positivity for S-100 protein, HMB45, and Melan A).

**Figure 5:**
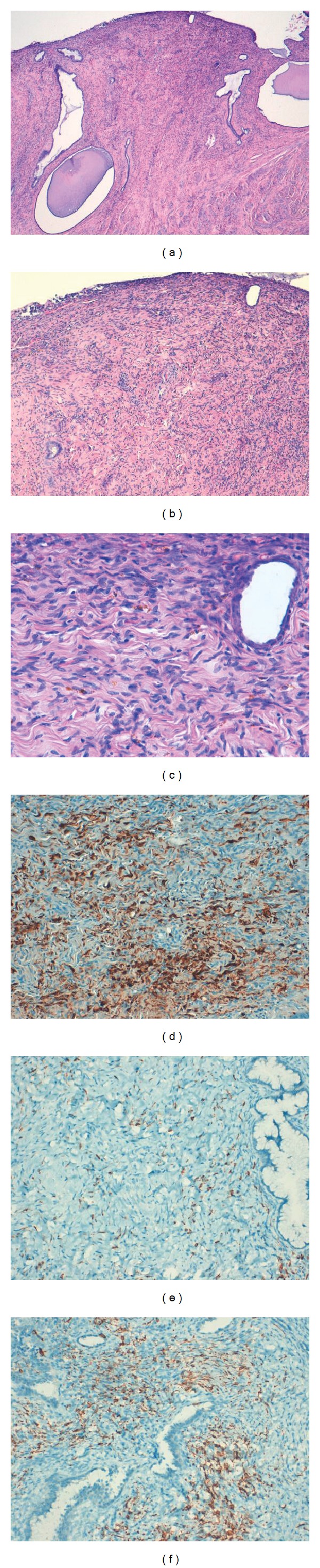
Uterine cervical blue nevi. Histopathologic findings. (a, b, and c) Upper stromal proliferation of focal pigmented melanocytes. The tumor cells are arranged in sheets and mostly spindle eosinophilic cytoplasm. Intracytoplasmic melanin is present. (d, e, and f) The tumor cells showed positivity for S-100, Melan A, and HMB-45, respectively.

In addition to previously described cervical lesion, a more or less symmetrical upper stromal proliferation of focal pigmented melanocytes was observed with an irregular contour and focal areas of pushing circumscribed border. The tumor cells were arranged in sheets. The tumor cells were mostly spindle-shaped with eosinophilic cytoplasm and contained intracytoplasmic melanin. The nuclei of the tumor cells were elongated with no or minimal irregularities of the nuclear membrane, evenly distributed chromatin, inconspicuous nucleoli, and no obvious mitotic figures. No dysplastic or atypical areas were seen. The tumor cells were positive for S-100, Melan A, and HMB-45 antigen (Figure 5).

The final diagnosis was malignant melanoma (7 mm and invasion level of 5 mm) and common blue nevi of the cervix. Lymph node affectation was also present.

## 3. Discussion

MM are generally found in areas of skin exposed to the sun, but can also involving mucosal membranes representing an exceedingly uncommon neoplasm which can occur in a variety of mucosal sites, such as the oral cavity, esophagus, anus and gynecological tract, among others [2, 10]. In particular, cervix MM, is more frequently founded in postmenopausal women over 50 years old [1]. The clinical presentation consists of vaginal bleeding or vaginal discharge of various degrees and duration, abdominal pain, dyspareunia, and postcoital bleeding [11, 12]. Our case showed similar previously reported clinical findings, characterized by postmenopausal presentation and abnormal vaginal bleeding.

Ethiopathogenesis of cervical uterine MM has not yet been completely elucidated. A possible hormonal influence in the development of cervical MM has been advocated [13]. Recently, a probable cofactor with human papillomavirus (HPV) infection and primary gynecologic MM has been pointed out [14]. In the study of Rohwedder et al. [14], they found the presence of HPV subtype 16 in two cases of vulvar MM. Therefore, it is likely that HPV could act either directly or indirectly on melanocytes in promoting cancer development [14]. Our study support this view. The p16 levels are increased in response to irregular cell cycle inactivation resulting from the disruption of interaction of pRb with transcription factor E2F in the presence of the HPV E7 oncogene [15, 16]. In addition, detection of p16 can serve as a surrogate biomarker for persistent infection with high-risk HPV [16]. We could demonstrate p16 at nuclear and cytoplasmic melanocytic neoplastic cells. In our case, the p16 expression could be related to high-risk HPV infection in malignant melanocytic cells, acting directly or indirectly in promoting uterine cervix malignant melanoma. Also the p16 expression, in our case, could be interpreting as concomitant finding with no implications in oncogenic mechanism of cervical malignant melanoma. No expression of p16 was observed in blue nevi cells.

Diagnosis of primary melanoma of the uterine cervix entertains a high probability of being confused with another entity, due to the rarity of the disease [17]. Differential diagnosis between a primary cervical melanoma and a metastatic tumor is important because the latter can be part of a metastatic disease spreading to the cervix [1, 3–6]. Based on data published by Norris and Taylor [18], the four criteria for diagnosis of primary malignant melanoma of the cervix include (a) presence of melanin in the cervical epithelium; (b) absence of melanoma in another site of the body; (c) presence of binding activity in the cervical epithelium near the lesion; (d) if metastatic disease is found, it should be according to the cervical carcinoma pattern. When strictly applying the criteria of Norris and Clark, only 23 published uterine cervix MM are founded in the literature [1]. In our case, we founded the four criteria of Norris and Taylor, with pagetoid dissemination to endocervical glandular epithelium, and metastatic cells at lymph node cervical carcinoma pattern.

An interesting topic is related to the evidence of melanocytic cells in the cervix. Three hypothesis have been proposed to try to understand the origin of melanocytes in the cervix: (a) melanocytic cells originating from Schwannian cells, (b) melanocyte migration from the neural crest, and (c) melanocytic differentiation from the epithelium of the endocervix wall [19, 20]. The most prevailing theory to explain the origin of blue nevi is that they arise from latent dendritic melanocytes from the embryologic migration of melanocytes from the neural crest [21]. Our case showed a coexisting malignant melanoma and common blue nevi of the cervix, this combination is a very rare association of two unusual melanocytic lesions at the uterine cervix [1, 22]. Only one previous case has been reported of combined melanocytic lesions at the female genital tract [23]. Our findings of a combination of melanocytic lesions in the uterine cervix, probably supported the notion of the embryologic migration of melanocytes from the neural crest.

In conclusion, we present a characteristic primary MM of the cervix combined with blue nevi. The MM is a rare disease with a poor prognosis, especially if it is not detected in a timely fashion or if it is not treated correctly, because it requires a different therapeutic approach and has a significantly worse prognosis. The differential diagnoses should include poorly differentiated squamous cell carcinoma, adenocarcinoma, rhabdomyosarcoma, and stromal sarcoma. No consensus has been established concerning treatment of primary melanoma of the cervix, but it is recommended to be surgical, procuring the establishment of 2 cm margins, accompanied by radio- or chemo-therapy.
